# Premedication with oral clonidine decreases intraoperative bleeding and provides hemodynamic stability in cesarean section

**DOI:** 10.5812/kowsar.22287523.1337

**Published:** 2011-07-01

**Authors:** Amin Ebneshahidi, Masood Mohseni

**Affiliations:** 1 Department of Anesthesiology, Sadi Hospital, Isfahan, IR Iran; 2 Department of Anesthesiology, Tehran University of Medical sciences, Tehran, IR Iran

**Keywords:** Clonidine, Cesarean Section, Blood Pressure, Hemodynamic

## Abstract

**Background:**

Intentional lowering of blood pressure helps to produce a desirably clean surgical field. Many drugs can be used to induce a hypotensive state, but due to their high potency and rapid effect, drugs that more easily and safely control the induction of hypotension are desirable.

**Objectives:**

To investigate the effects of premedication with oral clonidine on intraoperative bleeding and hemodynamic variables in patients undergoing cesarean sections.

**Patients and Methods:**

A total of 110 patients classified as American Society of Anesthesiologists (ASA) physical status I and II and who were scheduled for elective cesarean section under general anesthesia were enrolled. The patients were randomized to receive either oral clonidine (0.2 mg) or identical-looking placebo tablets 90 minutes before arriving at the operating room. Induction of anesthesia was performed by the same standard protocol in all patients. Systolic blood pressure (SBP) and diastolic blood pressure (DBP) and heart rate (HR) were recorded before and after induction; immediately after intubation; 5, 10, and 15 minutes thereafter; at the time of extubation; and one hour after the operation. The surgeons were asked to rate the quality of the operative field on a four-point scale that ranged from mild (1) to abundant bleeding (2).

**Results:**

Intraoperative SBP was lower in the clonidine group. After both intubation and extubation, the increases in SBP, DBP, and HR in clonidine-treated subjects were significantly less than the changes in the control patients. The amount of fentanyl given to control blood pressure and HR was significantly less in the clonidine group (18 ± 38 vs. 39 ± 53 μg, P = 0.02). The responses to the four-point scale indicated that intraoperative bleeding in the clonidine group was less than in the placebo group (1.2 ± 0.4 vs. 1.7 ± 0.6, P < 0.05).

**Conclusions:**

Premedication with oral clonidine can improve the hemodynamic management of cesarean cases.

## 1. Background

Intentionally lowering blood pressure is used to produce a desirably clean surgical field. Anesthesiologists have suggested many drugs, including volatiles, propofol, and remifentanil, for inducing a hypotensive state. In resistant cases, labetalol, nitroglycerin, and other peripherally acting vasodilators have been used to supplement the hypotensive action of first-line agents (1). However, the high potency and rapid effect necessitates invasive and tight hemodynamic monitoring to avoid severe hypotension during surgery. Therefore, the use of drugs that more are able to induce hypotension more easily and safely are desirable. In recent years, clonidine, a known antihypertensive agent, has generated considerable interest as an anesthetic adjuvant because its hypotension induction is more controllable for the central mechanism of action (2-5). Moreover, it has been suggested that it improves the hemodynamic stability after orotracheal intubation (6, 7). Interestingly, clonidine reduces requirements for anesthetics and enhances the effects of sedative, anxiolytic, and analgesic drugs (8-10). Most of prior trials on clonidine have focused on intraoperative intravenous administration of the drug (2, 11).

## 2. Objectives

We conducted a randomized, double-blind, placebo-controlled study to investigate the effects of oral clonidine premedication on intraoperative bleeding and hemodynamic variables in patients undergoing cesarean section.

## 3. Patients and Methods

At the time of this study, in the maternity wing of Sadi Hospital, 147 patients were classified as physical status I and II under the American Society of Anesthesiologists system and were scheduled for elective cesarean section under general anaesthesia. Of these patients, 110 patients responded that they were interested in participating in the study and were enrolled. After informed consent was obtained, the patients were randomized to receive either oral clonidine (0.2 mg) or identical-looking placebo tablets 90 minutes before arrival to the operating room. The randomization was performed by the hospital pharmacy by using a table of random numbers, and the patients, investigators, surgeons, and nurses involved in the patients’ care were blind to the assignment groups. Exclusion criteria were significant heart disease that contraindicated the use of controlled hypotension, medically important liver or kidney dysfunction, known allergies to clonidine, SBP > 160 mmHg, DBP > 90 mmHg, or pulse rate < 50 beats/min at the time of the preanesthetic visit. The institutional research ethics committee approved the study protocol.

### 3.1. Method of anesthesia

Patients were pretreated with dextrose-free crystalloids (500–700 mL) prior to induction. Induction of anesthesia was performed with thiopental 4 to 5 mg/kg and succinylcholine (1 to 1.5 mg/kg). Following tracheal intubation, anesthesia was maintained with 0.5% halothane delivered in a gas mixture of N2O (50%) and O2 (50%). After returning to spontaneous breathing, 0.5 mg/kg of atracurium was administered. Controlled mechanical ventilation with an initial tidal volume of 10 mL/kg and respiratory frequency of 10 breaths /min was adjusted to maintain normocapnia. After delivery, we continued to apply the volatile agent (halothane 0.8%) and administered 2 mg of morphine and 2 mg of midazolam, as well as increasing N2O concentration to 70%. Muscle relaxation was reversed with neostigmine (0.05 mg/kg) and atropine (0.02 mg/kg). Lactated Ringer’s solution was administered at a constant rate of 2 to 4 mL/kg/h using an infusion pump throughout the operation. The lowest MAP accepted was 60 mmHg. The desired control of MAP was attained with inspired concentration increments of halothane up to a maximum of 1 vol% as required. When it was unsuccessful, intravenous bolus of fentanyl (1 μg/kg) was administered. When hypotension occurred (MAP < 60 mmHg), fluid challenge (Ringer’s solution 10 mL/kg) and intravenous ephedrine (10 mg increments) were administered. During the operation, ECG monitoring, pulse oximetry, and capnometry were performed continuously.

### 3.2. Hemodynamic assessments

SBP and DBP as well as HR were recorded before and after induction; immediately after intubation and 5, 10, and 15 minutes thereafter; at the time of extubation; and also one hour postoperatively. After the operation, the surgeons were asked to rate the quality of the operative field on a four-point scale:

1 = bleeding, so mild it was not even a surgical nuisance;

2 = mild bleeding, a nuisance but without interference with accurate dissection;

3 = moderate bleeding that moderately compromised surgical dissection;

4 = massive bleeding that significantly interfered with dissection.

All the procedures were performed by the same team of surgeons.

### 3.3. Statistical analysis

Data were expressed as Mean ± standard deviation. Quantitative variables were compared with two-sample independent t tests, and nominal data were analyzed with χ2 tests. Within-group comparisons of hemodynamic parameters were performed with paired-sample t tests. P < 0.05 were considered statistically significant. Statistical analyses were performed using the Statistical Package for Social Sciences software, version 11.0 (SPSS Inc., Chicago, IL).

## 4. Results

Patients’ characteristics (Table 1) and hemodynamic parameters at the time of admission (Table 2) were not statistically different between the clonidine and placebo groups. Assessment of hemodynamic parameters in the operating room before induction of anesthesia showed that SBP and DBP but not HR in the clonidine group were significantly lower than in the control group. Moreover, SBP in 5-minute interval assessments in the intervention group was significantly less than in the placebo group. At recovery, blood pressure and HR measurements in the clonidine group were less than the placebo group (Table 2). Within-group comparisons showed that in the clonidine-treated patients, HR before and after induction was not statistically different (P > 0.05). Also the decrease in blood pressure after induction in the clonidine group was less than in the placebo group (SBP 3 vs. 7 mmHg; DBP 5 vs. 9 mmHg, P < 0.05). The results showed that after both intubation and extubation, the increases in SBP, DBP, and HR in clonidine-treated subjects were significantly lower than the changes in the control patients. The amount of fentanyl given to control blood pressure and HR was significantly less in the clonidine group (18 ± 38 vs. 39 ± 53 μg, P = 0.02). The frequency of fluid challenge for the management of intraoperative hypotension was not statistically different between the clonidine and placebo groups (9.1% vs. 12.7%, respectively; P > 0.05). One patient in the placebo group needed additional ephedrine administration to control hypotension. Significant bradycardia was not observed in our series. According to the results of the four-point scale, intraoperative bleeding in the clonidine group was, on average, less severe than in the placebo group (1.2 ± 0.4 vs. 1.7 ± 0.6, P < 0.05).

**Table 1.** Comparison of Patient Characteristics and Intraoperative Conditions

Variable	Clonidine (n = 55)	Placebo (n = 55)	P value
**Age (y) (Mean ± SD)**	25.6 ± 4.3	24.8 ± 4.4	0.31
**Weight (Kg) (Mean ± SD)**	66.7 ± 8.7	67.1 ± 9.4	0.56
**Height (Cm) (Mean ± SD)**	161.4 ± 9.3	162.6 ± 10.5	0.72
**Anesthesia ** ^**a**^ ** (min) (Mean ± SD)**	41.2 ± 3.2	41.9 ± 3.6	0.64
**Surgery (min) (Mean ± SD)**	32.1 ± 2.7	32.7 ± 2.9	0.69
**Fluid bolus [No. (%)]**	5 (9.1)	7 (12.7)	0.76

^a^ Minutes from induction to when the patient’s eyes open

**Table 2.** Hemodynamic Parameters in Clonidine-Treated Patients and Controls

Hemodynamic variables	SBP ^a^		DBP ^b^		HR ^c^	
	Clonidine	Placebo	Clonidine	Placebo	Clonidine	Placebo
**Admission (Mean ± SD)**	117 ± 18	118 ± 17	72 ± 14	73 ± 14	85 ± 13	83 ± 13
**Pre-induction (Mean ± SD)**	114 ± 17	123 ± 19 ^d^	66 ± 13	77 ± 15 ^d^	92 ± 14	96 ± 14
**Post-induction (Mean ± SD)**	111 ± 15	116 ± 19	61 ± 13	69 ± 14	89 ± 14	89 ± 15
**After orotracheal intubation (Mean ± SD)**	117 ± 16	129 ± 18 ^d^	65 ± 13	77 ± 15 ^d^	94 ± 15	108 ± 16 ^d^
**5 min after the incision (Mean ± SD)**	104 ± 15	111 ± 19 ^d^	61 ± 14	64 ± 15	82 ± 13	88 ± 14
**10 min after the incision (Mean ± SD)**	99 ± 18	107 ± 17 ^d^	58 ± 13	63 ± 14	76 ± 12	82 ± 13
**15 min after the incision (Mean ± SD)**	97 ± 15	109 ± 18 ^d^	59 ± 13	61 ± 14	74 ± 13	79 ± 14
**Extubation (Mean ± SD)**	104 ± 19	121 ± 18 ^d^	62 ± 14	71 ± 15 ^d^	91 ± 15	104 ± 15 ^d^
**One hour after operation (Mean ± SD)**	112 ± 16	119 ± 17 ^d^	62 ± 11	71 ± 13 ^d^	78 ± 14	92 ± 15 ^d^

^a^ SBP = systolic blood pressure

^b^ DBP = diastolic blood pressure

^c^ HR = heart rate

^d^ P < 0.05 in two-sample independent t-test

## 5. Discussion

Although clonidine has been used successfully as an adjunct for hypotensive techniques (2-5), another study found a contradictory effect of clonidine on intraoperative bleeding (12). Comparable to our findings, a study on 40 patients undergoing middle-ear surgery showed that clonidine improved the operative field (13). Most studies with negative findings had kept the arterial pressure of patients in a narrowly defined range, and thus those analyses were unlikely to reveal the beneficial effects of clonidine on surgical site bleeding. The present study demonstrated the favorable effects of premedication with oral clonidine on blood loss and the quality of the surgical field during cesarean section. Endotracheal intubation during anesthesia increases blood pressure and HR. Previous research has suggested that such hemodynamic changes have little consequences in otherwise healthy young adults (14). However, patients with certain comorbidities such as hypertension, cardiovascular, or renal disease and complicated pregnancies who are candidates for cesarean section may not tolerate such hemodynamic fluctuations. Clonidine as an α2–agonist has been shown to reduce sympathetic outflow (15). For clinical implications, the results of this study demonstrate that clonidine improves the hemodynamic stability of patients after both endotracheal intubation and extubation. The importance of the use of clonidine in high-risk patients needs further investigation. Previous studies have suggested that preoperative treatment with clonidine reduces the total requirement of other medications to maintain the blood pressure in the target range (16, 17). Similarly, lower fentanyl requirements in our clonidine-treated patients as well as our finding of decreased intraoperative SBP indirectly support the results of previous studies. On the other hand, uncontrolled hypotension and bradycardia are the major potential adverse effects of clonidine that may be encountered in intraoperative intravenous use of clonidine (18-20). In this study, the requirement for fluid challenge was comparable between the two groups, and no case of significant bradycardia was observed, which supports the safety of premedication with oral clonidine. In summary, oral premedication with clonidine safely reduces intraoperative blood pressure and as a result minimizes intraoperative blood loss. In addition, clonidine reduces the total requirement for other anesthetics and lessens the unwanted hemodynamic response to endotracheal intubation and extubation. Further studies are needed to find the optimal dose, the most appropriate time for administration, and the most effective route of administration of clonidine.
